# Emmenological Questions

**Published:** 1839-01

**Authors:** 


ART. X. — *Questiones Emmenologicas, scripsit* JOH. FRIED. KRIEG, Med. et Chir. Doctor apud Martisburgenses practicus.—*Lipsiæ*, 1838. 4to. pp. 30.

*Emmenological Questions.* By JOHN FREDERICK KRIEG, M.D. et Chir. —*Leipsig.*

THE work which stands at the head of this notice belongs to a class for which writers, we are happy to say, are not likely to be found in this country; at least, not amongst those who legitimately practise the profession of medicine. There can be no doubt that works replete with indelicacy, and published under the denomination of medical books, are frequently issued from the British press; but of these professional men know nothing, except from the public notices which so incessantly are obtruded before his eye. They in nowise form a portion of his library. They are the odious productions of sordid, wicked, and ignorant charlatans. On the continent, however, works, sometimes made up of details of the most disgusting nature are not unfrequently published by those who rank with scientific men, and whose education has rendered them well qualified to practise the honorable art of medicine. The author of these Emmenological Questions belongs to this class: he is a learned doctor in surgery and medicine, and therefore has some claim upon our notice. As journalists, we give an analysis of his pages, but forbear making any comment upon them: as he has written, so we render.

Dr. Krieg addresses himself to three different subjects, and endeavours to show, 1st, that the menstrual fluid is not a secretion of the uterus, but of the vagina; 2d, that the halitus which is exhaled from the person of a healthy female during menstruation, as likewise the menstrual fluid itself, is the most natural and best emmenagogue; 3d, that the chenopodium olidum, which has an odour resembling that of the menstrual fluid, is not an inefficacious substitute.

I. In support of the first position, Dr. K. states (1) that, in 1829, he examined the body of a woman of forty years of age, who had menstruated only a few days before death, in which case the cavity of the uterus was obliterated by cancer, and that it had neither neck nor orifice; (2) that a blenorrhœa of the uterus can be seen, by aid of the *speculum vaginæ*, proceeding from that organ, and which has never been observed to be the case with the menstrual secretion; (3) that the uterus has never been known to be distended by retention of the menstrual discharge; (4) that gravid women have been known to menstruate, and that without injury to the fœtus, which he conceives could not have been, did the discharge proceed from the uterus; and he states the case of a female who was at all times amenorrhœal, excepting during pregnancy, when she regularly menstruated. He relates other cases to support his view, and quotes Joh. Bohnius, who dissected a female that was strangled during the period of the catamenial discharge, in whom the uterus was found dry, while the vagina was bedewed with menstrual fluid. Dr. K. does not altogether deny that there may be a stillicidium of blood from the uterus; but he contends that the chief and proper seat of the menstrual secretion is the vagina, and that, whatever blood may be voided by the uterus, with the exception of the puerperal lochial discharges, is to be esteemed an hemorrhage, and not a secretion. He then enters into a description of the membrane of the vagina, whose structure, he says, plainly points it out as the secreting organ. He condemns frequent washings and excessive cleanliness of these parts, affirming that the emunctories are thus prematurely closed, when regurgitations of excrementitious humours takes place, and in this way producing many diseases, a long list of which he specifies,—as ovarian tumours, scirrhous affections, hysteria, diabetes, neuralgia of the abdominal ganglia, &c.; and which diseases he states most frequently to occur in meretricious women.

II. In reference to the second question, which is the chief purport of his essay, Dr. Krieg notices the peculiar odour which emanates from females at the period of puberty; and says that, as the *fomes* of diseased states are the source of similar diseases in those who may come in contact with them, so, in like manner, is the fœtor exhaled during the menstrual period capable of producing the catamenial state in those in whom the secretion has been suppressed. He first speaks of the efficacy, as an emmenagogue, of eating (fasting) well-fermented bread embued with the halitus! This impregnation is to be effected by its being worn in the linen of a female during the catamenial period! He quotes Hoffmann as not blushing to recommend the practice. "Hot bread," says this author, "taken from the furnace, and carried about a female when under the influence of menstruation, remedies suppression of the menses, if it be given to the extent of half a drachm or a drachm, dried and then soaked in wine; as likewise clean linen, dipped in the menstrual blood of the virgin sister, and dried and infused in hot wine, which, when cooled, is to be taken early in the morning; also the being clothed in an under-shift imbued with the recent discharge, (especially if of one near of kin,) is proved by experience to excite the discharge when suppressed." (*Clavis Schrœderiana. Halæ*, 1681.) Dr. Krieg, however, thinks that these different modes may be nauseous and disagreeable, and having discovered that the halitus exhaled during menstruation is equally efficacious, strongly recommends that the amenorrhœal female should be placed in bed with a healthy virgin during the period of secretion; and he states, with Hoffmann, that he has found that the more certain effect is produced by a sister or one near of kin. In support of these views he relates some cases, in all of which cures were effected, though other means had been resisted. He also recommends inoculation, which is done by introducing *per vaginam* a sponge or lint dipped into the fluid! Dr. K., however, appears to have some misgivings as to the delicacy of his remedy, and tells us, in extenuation, that it has been formerly used by some of the soberest of men, both in the form of tincture, draught, and powder; and especially quotes Daniel Ludovic, physician royal at Saxe Gotha, as recommending and employing it, and as also regretting the disuse into which it had fallen from being esteemed indecent: this, Dr. K. says, it cannot be considered to be, when used by prudent and chaste men: nor can he think it dirty (*sordidus*), if taken from a healthy virgin; nor nauseous, if it be administered to one unconscious of what they take; and, lastly, not indecent (*impius*), if from this remedy health be procured!

III. The concluding portion of this dissertation is taken up with a statement of the efficacy of the chenopodium olidum* in amenorrhœa. He was induced to test its powers from its fœtor so greatly resembling the halitus of the female during menstruation, and he found it answer his warmest expectations. This herb, he says, may be administered either by itself or with other medicines in torpor and obstruction of the uterine system; adding that its efficacy is attributable to the solvent, repellent, aperient, and excitant properties which it possesses. Its first effect is that of setting free the suppressed menses, and then of subduing the menstrual colic and neuralgia of the abdominal ganglia: it also relieves many hysterical conditions, and he thinks that it may be useful to excite the venereal stimulus. He prefers for use the recently expressed juice, either exhibited alone or mixed with wine or spirit, or with the syrups of orange or rhubarb, or (what is more preferable) the syrups of wormwood. By keeping or drying, this plant loses the greater part of its specific odour; and its efficacy is quite destroyed by decoction or extraction. In conclusion, he details two cases in which it proved eminently successful.

* Chenopodium olidum, *seu* Vulvaria, stinking goosefoot: it occurs in waste ground, especially among sand or rubbish near the sea.

